# Connections between Transcription Downstream of Genes and cis-SAGe Chimeric RNA

**DOI:** 10.3390/genes8110338

**Published:** 2017-11-22

**Authors:** Katarzyna Chwalenia, Fujun Qin, Sandeep Singh, Panjapon Tangtrongstittikul, Hui Li

**Affiliations:** 1 Department of Pathology, School of Medicine, University of Virginia, Charlottesville, VA 22908, USA; kmc4fv@virginia.edu (K.C.); fq9m@virginia.edu (F.Q.); ss7mh@virginia.edu (S.S.); pt5fn@virginia.edu (P.T.); 2 Department of Biochemistry and Molecular Genetics, School of Medicine, University of Virginia, Charlottesville, VA 22908, USA

**Keywords:** DoG, Cis-SAGe, chimeric RNA, osmotic stress

## Abstract

cis-Splicing between adjacent genes (cis-SAGe) is being recognized as one way to produce chimeric fusion RNAs. However, its detail mechanism is not clear. Recent study revealed induction of transcriptions downstream of genes (DoGs) under osmotic stress. Here, we investigated the influence of osmotic stress on cis-SAGe chimeric RNAs and their connection to DoGs. We found, the absence of induction of at least some cis-SAGe fusions and/or their corresponding DoGs at early time point(s). In fact, these DoGs and their cis-SAGe fusions are inversely correlated. This negative correlation was changed to positive at a later time point. These results suggest a direct competition between the two categories of transcripts when total pool of readthrough transcripts is limited at an early time point. At a later time point, DoGs and corresponding cis-SAGe fusions are both induced, indicating that total readthrough transcripts become more abundant. Finally, we observed overall enhancement of cis-SAGe chimeric RNAs in KCl-treated samples by RNA-Seq analysis.

## 1. Introduction

Chimeric RNAs involving transcripts derived from two neighboring genes are a well-known phenomenon [1,2,3,4,5,6,7,8,9,10,11,12,13], and they are estimated to comprise roughly 5% of the human transcriptome [12]. Multiple terms have been given to these chimeras: transcription induced chimeras [1,2,3,4,5], tandem RNA chimeras [6], conjoined genes [7,8] and read-through fusions [9]. We prefer to use the term cis-splicing of adjacent genes (cis-SAGe), as it clearly distinguish from RNA trans-splicing. Till now, the molecular basis of cis-SAGe chimeric RNA formation remains elusive. Several ideas have been proposed [8,14,15,16]. Intuitively, most of them imply transcriptional readthrough the gene boundaries between two neighboring loci. Omitting transcription termination signal is well known in lower organisms [17,18]. Recently it had also been shown to occur in human ovary cells [16]. Interestingly, under conditions of osmotic stress, transcripts reading through the gene boundaries seems to be more profound as recently reported by Vilborg et al. [19]. In their work, they describe a new class of transcripts called “DoGs” containing transcripts downstream of the genes. We asked the question whether cis-SAGe chimeric RNAs are induced under osmotic stress conditions, and whether the DoGs are relevant to the formation of cis-SAGe chimeric RNAs.

## 2. Materials and Methods

### 2.1. Cell Culture and Osmotic Stress Onduction

HEK293T cells were maintained in Dulbecco’s Modified Eagle’s Medium with 4500 mg/L glucose (Gibco) supplemented with 10% fetal bovine serum (FBS) and 1% Pen/Strep solution (Hyclone). Cells were cultivated at 37 °C in 5% CO2 humidity. For osmotic stress induction, cells were counted and seeded 24h before experiment. Stress was induced by adding 1 M solutions of KCl, NaCl, or Sucrose in medium to obtain final concentration of 80 mM (for KCl and NaCl) or 200 mM (for Sucrose).

### 2.2. RNA Extraction

RNA was extracted from cell lines using TRIzol Reagent (Life Technology, Carlsbad, CA, USA) according to manufacturer’s instruction. RNA samples were analysed on a NanoDrop (Thermo Scientific, Waltham, MA, USA) and 3 μg RNA was used for cDNA synthesis. All RNA samples in this study were treated with DNAse I (NEB, Ipswich, MA, USA), followed by standard Reverse Transcription using SensiFAST cDNA Synthesis Kit (Bioline, Boston, MA, USA) according to manufacturer’s instructions.

### 2.3. Quantitative Reverse Transcription Polymerase Chain Reaction (qRT-PCR)

All primers used in this study (listed in Table S1) were designed using Primer3 software (http://primer3.ut.ee/) and synthesized by Eton Bioscience Inc., Research Triangle Park, NC. Primers for DoGs were designed within 1000 bp–1500 bp downstream of AATAAA (poly-A) site. If AATAAA was not found in genomic sequence, primer was designed within 1000 bp–1500 bp downstream of the end of last exon.

Step One Plus Real-Time PCR System (Applied Biosystems, Foster City, CA, USA) was used to perform SYBR Green based qPCR experiments. Relative RNA levels were calculated using 2^−(ΔΔCt)^ method. Tested genes were normalized to GAPDH (glyceraldehyde 3-phosphate dehydrogenase) gene.

### 2.4. Statistics

Evaluation of significance was performed using *t*-test. Correlation between fusion and DoG of the 5’ gene was calculated using Pearson Correlation function.

### 2.5. Bioinformatics

We downloaded raw RNA-Seq data from study SRP058633, which contains three biological replicates of untreated cells or cells treated with 80 mM KCl for 1 h. Software Ericscript was used to identify candidate chimeric RNAs. A cut off of Ericscore at 0.5 was used. We then selected chimeric RNAs that were at least duplicated in each group.

## 3. Results

### 3.1. cis-SAGe Chimeric RNAs and Corresponding DoGs Are not All Induced after 1 h Treatment

**Figure 1:**
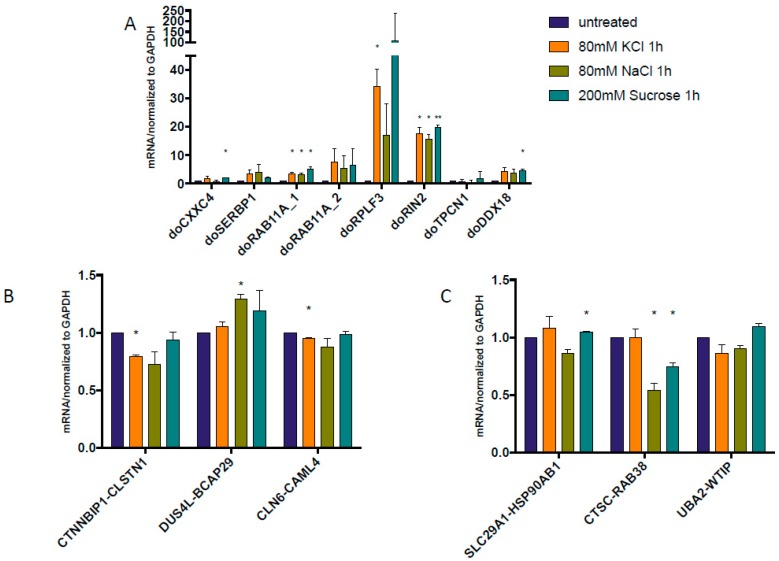
Quantitative reverse transcription polymerase chain reaction (qRT-PCR) measuring the levels of downstream of genes (DoGs) and cis-splicing between adjacent genes (cis-SAGe) fusions at 1 h after osmostress. RNAs were extracted from HEK293T cells treated or untreated with three osmotic stresses. (**A**) DoGs from the previous report [19] were measured, and confirmed their induction after the stress; (**B**) Three cis-SAGe fusion RNAs, *CTNNBIP1-CLSTN1*, *DUS4L-BCAP29*, and *CLN6-CALML* were measured; (**C**) Addition cis-SAGe RNAs, *SLC29A1-HSP90AB1*, *CTSC-RAB38*, and *UBA2-WTIP*, were selected based on the discovery of DoGs of their 5’ parental genes. The levels of various transcripts were normalized to that of *GAPDH*, and further normalized to the untreated samples. *: *p* < 0.05, **: *p* < 0.01.

We used the same osmotic stresses as described before [19]. In order to confirm the induction of gene read-through by osmotic stress, we measured the expression level of previously described DoGs. Indeed, after 1 h treatment, higher expression levels of these DoGs were found with KCl, NaCl, or sucrose treatment, except for doTPCN1 (Figure 1A). We then checked the expression of several confirmed cis-SAGe chimeric RNAs: *CTNNBIP1-CLSTN1* [20], *DUS4L-BCAP29* [21,22], and *CLN6-CALML* [11]. Only *DUS4L-BCAP29* had slightly induction after 1 h NaCl treatment (Figure 1B). Other chimeras seem to remain unchanged or were even downregulated by the 1 h osmotic stresses. Suspecting that these cis-SAGe fusions may not be the best models to study the effect of osmotic stress and DoGs, we data-mined the list of DoGs reported previously [19] and identified several cis-SAGe fusion RNAs that have DoGs from their 5’ parental genes. These are also cis-SAGe chimeras we validated before: *SLC29A1-HSP90AB1* [11], *CTSC-RAB38* [20,22], and *UBA2-WTIP* [23]. However, when we examined the response of these fusion RNAs under the same osmotic stress conditions, we observed the same phenomenon (Figure 1C), i.e., the fusion RNAs remained largely unchanged or even downregulated in most situations.

### 3.2. Some DoGs and Their Corresponding Chimeric RNAs Correlate Negatively

**Figure 2:**
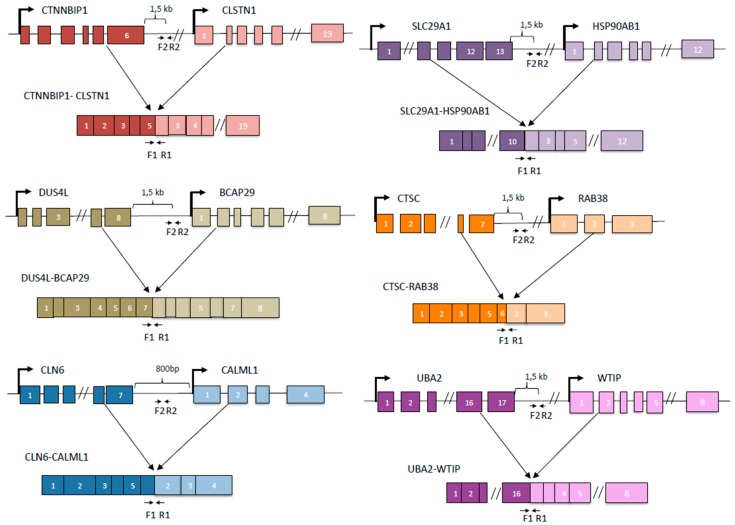
Cartoons depicting the configurations of cis-SAGe fusion RNAs and primer locations. For each fusion, upper level is the DNA configuration. Lower level is the chimeric RNA configuration. Primer set 1 (F1 and R1) is used to amplify the chimeric RNA. Primer set 2 (F2 and R2) is used to amplify DoG of the 5’ parental gene.

**Figure 3:**
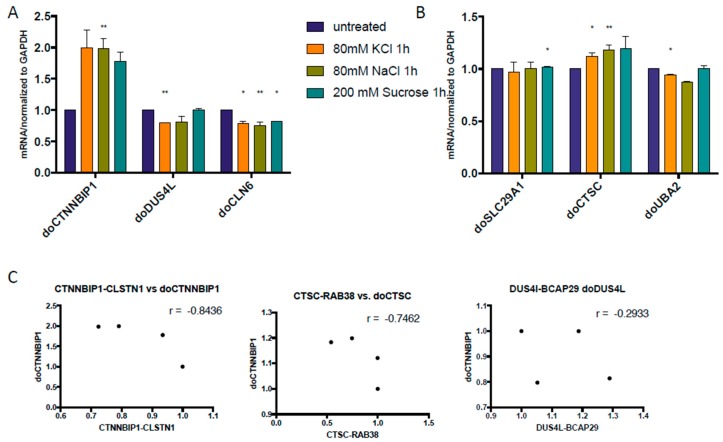
Inverse correlation between DoGs and corresponding cis-SAGe RNAs at 1 h after osmostress. (**A**) DoGs of the 5’ parental genes of *CTNNBIP1-CLSTN1*, *DUS4L-BCAP29*, and *CLN6-CALML* were measured; (**B**) DoGs of the 5’ parental genes of *SLC29A1-HSP90AB1*, *CTSC-RAB38*, and *UBA2-WTIP* were measured. The levels of various transcripts were normalized to that of *GAPDH* and further normalized to the untreated samples; (**C**) The levels of the DoGs and their fusion RNAs were plotted in scatter plots. Three examples are shown here. *: *p* < 0.05, **: *p* < 0.01.

We then designed primers and use quantitative PCR to evaluate the response of the DoGs of the corresponding cis-SAGe fusions. To do so, we searched for consensus 5’-AATAAA-3’ (polyadenylation signal). Real-time PCR primers were designed within 1000 bp–1500 bp downstream of Poly A signal. If AATAAA was not found in the genomic DNA sequence, we used the end of last exon (Figure 2). We treated all RNAs with DNaseI to get rid of genomic DNA contaminants. When we measured these DoGs of the 5’ parental genes of our cis-SAGe fusion transcripts, we noticed much less dramatic changes compared with the DoGs in Figure 1A. We did observe some induction of DoGs for *CTNNBIP1* and *CTSC* genes (Figure 3A,B). Interestingly, their corresponding fusion RNAs, *CTNNBIP1-CLSTN1*, and *CTSC-RAB38* had some level of downregulation under the osmotic stresses (Figure 1B,C). Conversely, we observed slightly upregulation of *DUS4L-BCAP29* fusion RNA (Figure 1B), and slight downregulation of the DoG of *DUS4L* gene (Figure 3A).

We suspected that during the initial phases after osmotic stress, at least for some cis-SAGe fusion RNAs, the amount of precursor mRNAs may be limited, which causes competition between the DoG and fully formed chimeric RNAs. In these situations, if more DoGs are present, the level of mature chimeric RNAs will be reduced, and vice versa. To test this hypothesis, we checked correlation between the chimeric RNAs and corresponding DoG in samples where we could see obvious changes exerted by the osmostress (Figure 3C). Indeed, there is an inverse correlation between the chosen chimeras and their corresponding DoGs, supporting the competition theory.

### 3.3. Both DoGs and Corresponding cis-SAGe Fusions Are Induced in Response to Prolonged Treatment

**Figure 4:**
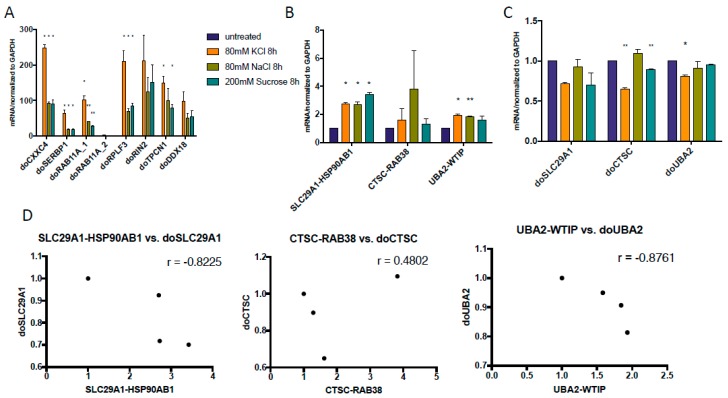
DoGs and corresponding cis-SAGe RNAs at 8 h after osmostress. (**A**) Three cis-SAGe fusion RNAs, *SLC29A1-HSP90AB1*, *CTSC-RAB38*, and *UBA2-WTIP*, were measured; (**B**) Their corresponding DoGs from the 5’ parental genes were measured. The levels of various transcripts were normalized to that of *GAPDH*, and further normalized to the untreated samples; (**C**) The levels of the DoGs and their fusion RNAs were plotted in scatter plots. *: *p* < 0.05, **: *p* < 0.01.

The dynamic between cis-SAGe fusion RNAs and DoGs may change as the cells expose to longer osmostress. We then tested the previously published DoGs [19] at 8 h time point and found that their induction was even more dramatic at 8 h (Figure 4A) than at 1 h time point (Figure 1A). When we examined the three cis-SAGe fusions with DoGs extracted from the same dataset, we noticed some induction of *SLC29A1-HSP90AB1* and *UBA2-WTIP* (Figure 4B). However, the corresponding DoGs of the 5’ parental genes were still unchanged or downregulated (Figure 4C). Similar to 1 h, there seem to be an inverse correlation between some of the DoGs and their corresponding cis-SAGe fusion RNAs (Figure 4D).

**Figure 5:**
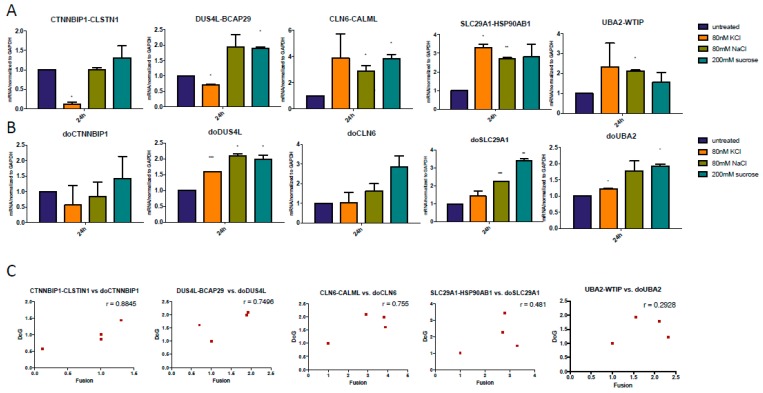
Positive correlation between DoGs and corresponding Cis-SAGe RNAs at 24 h after osmostress. (**A**) cis-SAGe fusion RNAs were measured. Most of them were induced by osmostress; (**B**) DoGs of the 5’ parental genes of the fusions were measured. Most of them were also induced by osmostress. The levels of various transcripts were normalized to that of *GAPDH* and further normalized to the untreated samples; (**C**) The levels of the DoGs and their fusion RNAs were plotted in scatter plots. *: *p* < 0.05, **: *p* < 0.01, ***: *p* < 0.001.

However, at 24 h time point, cis-SAGe chimeric RNAs were upregulated by osmotic stress in nearly all cases (Figure 5A). The corresponding DoGs were also induced (Figure 5B). Interestingly, the levels of the fusion and DoGs were positively correlated (Figure 5C). These results are consistent with the idea that over time, more transcriptional readthrough occurs, with some remaining as DoGs, and some processed into cis-SAGe fusions.

We then examined the presence and strength of polyadenylation signals in the 5’ parental genes. Within 40bp after the last exon, we found classic polyadenylation signal 5’-AATAAA-3’ in only *UBA2* gene. We found 5’-ATTAAA-3’ (about 77% strength of 5’-AATAAA-3’ [24] in *DUS4L* and *CTSC* genes. We also found 5’-AGTTAA-3’ in *CLN6* has (about 29% strength of 5’-AATAAA-3’ [24]). Interestingly, we did not find any of the three most common polyadenylation signals in *CTNNBIP1* or *SLC29A1*. Therefore, no obvious correlation between the presence and type of polyadenylation signal, and chimeric RNA induction was observed.

### 3.4. The Upregulation of DoGs and cis-SAGe Fusions Persists after Osmotic Stress

**Figure 6:**
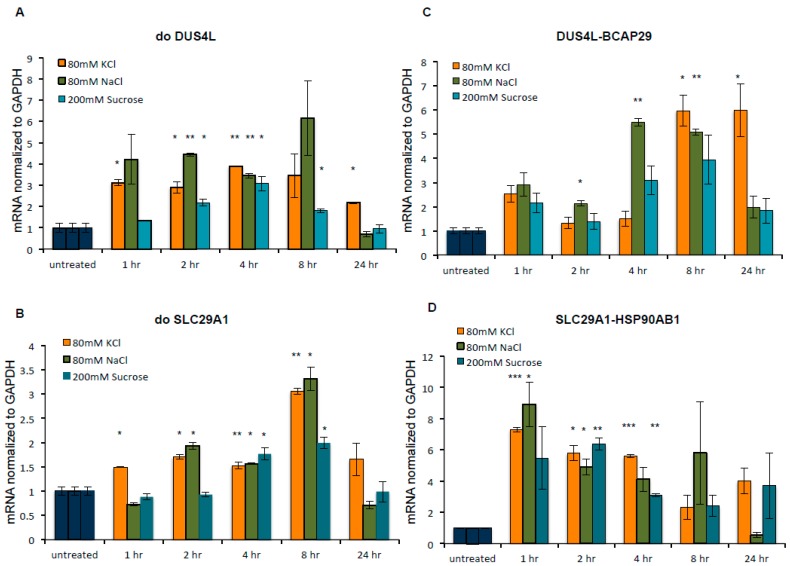
The upregulation of DoGs and cis-SAGe fusions persisted after osmostress. HEK293 cells were first treated with KCl, NaCl, or sucrose for 24 h to induce changes in DoGs and fusion RNAs. The stress was then removed, and time points were collected at 1 h, 2 h, 4 h, 8 h, and 24 h after the wash-off. DoGs and cis-SAGe fusion RNAs were measured by qPCR. The levels of various transcripts were normalized to that of *GAPDH* and further normalized to the untreated samples. Examples of DoGs, doDUS4L (**A**) and doSLC29A1 (**B**), as well as representative cis-SAGe fusion RNAs *DUS4L-BCAP29* (**C**), and *SLC29A1-HSP90AB1* (**D**) are shown here. *: *p* < 0.05, **: *p* < 0.01, ***: *p* <0.001.

To determine the reliance of the DoGs and cis-SAGe fusion to the osmotic stress, we conducted a wash-off experiment. HEK293 cells were first treated with KCl, NaCl, or sucrose for 24 h to induce changes in DoGs and fusion RNAs, followed by removing the stress and collecting cells at 1 h, 2 h, 4 h, 8 h, and 24 h time points after the wash-off. We found that most DoGs and cis-SAGe fusion levels continued to increase even after the stress conditions were removed, and the upregulation persisted longer than eight hours (examples in Figure 6). Different stress conditions and different transcripts varied in the time that the trend persisted.

### 3.5. KCl-Induced Osmostress Increases the Global Occurrence of cis-SAGe Chimeric RNAs

**Figure 7:**
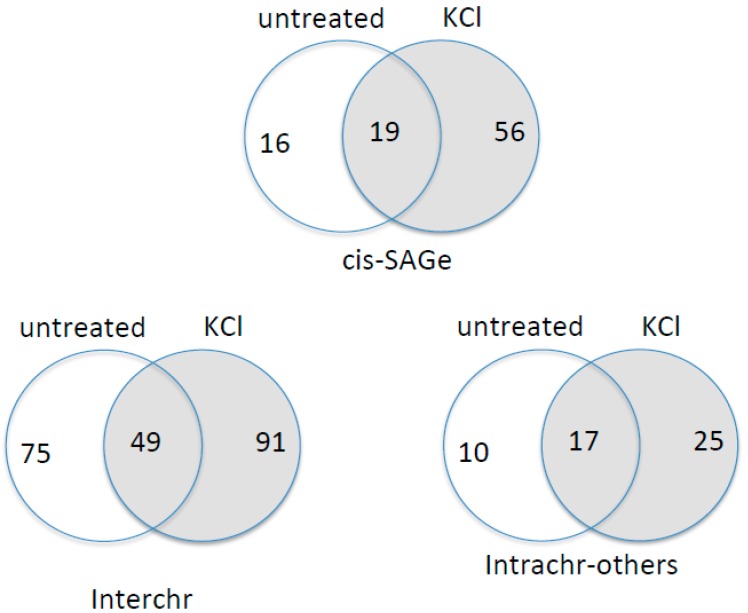
RNA-Seq analyses of chimeric RNAs with or without KCl-induced osmotic stress. Chimeric RNAs were grouped into three categories: cis-SAGe, Interchr, and Intrachr-others. Venn grams demonstrate the common and unique chimeric RNAs in each sample group.

Finally, we examined global chimeric RNA changes in the cells under osmotic stress. Using Ericscript software [25], we analysed raw RNA-Seq data, which includes triplicate of KCl-treated and untreated cells [19]. We selected chimeric RNAs that were at least duplicated in each group. We then binned the chimeric RNAs into three categories: parental genes from different chromosomes (Interchr), the same chromosome and between adjacent, same strand genes (cis-SAGe), or the same chromosome, opposite strand, or not adjacent genes (Intrachr-others) [11,20] (Figure 7). The most obvious change is the increased number of cis-SAGe fusions in KCl treated group.

## 4. Discussion

Previously, chimeric RNAs, which are composed from fragments derived from two separate genes, were mainly ascribed to cancer cells and were believed to be produced as a result of chromosomal rearrangements [26,27,28,29]. However, there is more and more evidence of chimeric RNAs in non-cancer tissues and cells [20,30,31,32,33]. We now know that chimeric RNAs can also be formed via either trans-splicing [34,35,36,37,38,39] or cis-SAGe [10,11,12,14]. Molecular mechanism of these two processes is still not well defined. For the latter, cis-SAGe chimeras are likely formed due to omitting transcription termination signal of the upstream gene. This phenomenon is well known in lower organisms as “transcription antitermination” [17,18] and was confirmed in rat cells [15] and more recently in human ovarian cells [16].

Interestingly, a recent study published by Vilborg et al. showed that omitting transcription termination signal occurs in normal conditions at very modest levels, but is greatly induced by hyperosmotic stress [19]. Transcripts are created at up to several dozens of kb downstream of parental genes and are named DoGs [19,40]. Significant induction of chosen DoGs can be observed as soon as 1 h after osmotic stress induction and is even greater after 8 h of treatment [19] (Figure 1A and Figure 4A). Since the distance between neighboring genes that form cis-SAGe fusions falls within the range of 8.5–30 kb [13], it is possible that some of the DoGs may actually contribute to the formation of cis-SAGe chimeric RNAs.

However, we observed no obvious changes and even slight downregulation with several of the confirmed cis-SAGe chimeric RNAs at 1hr time point. At 8 h time point, several fusions were induced. Even though we could detect transcripts downstream from the 5’ parental genes, they were not as dramatically induced by the osmostress at early time points (1 h and 8 h) as the examples given in the report by Vilborg et al. [19]. We also observed an inverse correlation between the DoGs and their corresponding cis-SAGe fusions. At 24 h, both DoGs and cis-SAGe fusions were induced, and their expression levels had a positive correlation. These results are consistent with a model that at early time point of osmotic stresses, the transcripts passing through the 5’ gene boundary are about the same level as untreated cells. When they are processed into mature cis-SAGe fusions, the level of DoGs will be reduced. However, at later time point, the total amount of the readthrough transcripts are increased, which are manifested by more DoGs and more processed cis-SAGe fusion RNAs.

It is possible that there are two groups of DoGs. One group has rapid and dramatic responses as the ones shown in Figure 1A (ten to hundreds of fold inductions at early time points). If they have corresponding cis-SAGe fusions, we anticipate a dramatic induction even at 1 h time point. The 5’ genes of the cis-SAGe fusion RNAs enriched in KCl treated samples (Figure 7) probably belong to this group. The other group has slow and less dramatic responses, such as the ones we tested in Figure 3 and Figure 4. The limited total pool of readthrough transcripts causes a direct completion of DoGs and corresponding cis-SAGes.

Different fusions had different responses to osmostress. While there was an inverse correlation between *CTSC-RAB38* and doCTSC at 1 h, this changes to a positive correlation at 8 h of treatment. On the other hand, *SLC29A1-HSP90AB1* and *UBA2-WTIP* correlate negatively with their DoGs at even 8 h. These differences indicate high variability in osmotic stress response among individual chimeric RNAs. This may be due to different strength of polyadenylation sites (as observed by Vilborg et al. [19]). In addition, we also noticed variable among different osmotic stresses. Moreover, it was shown that different cell lines have different pattern of response to hyperosmotic conditions [41].

## Supplementary Materials

The following are available online at www.mdpi.com/2073-4425/8/11/338/s1.
