# Supplementary material for: Connections between Transcription Downstream of Genes and cis-SAGe Chimeric RNA
Source: Genes (Basel). 2017 Nov 22;8(11):338. doi: 10.3390/genes8110338 (PMC5704251; doi:10.3390/genes8110338)
Supplement: Supplementary file 1 [file genes-08-00338-s001.pdf]

Figure S1: Gel-electrophoresis picture of representative cis-SAGe fusions.

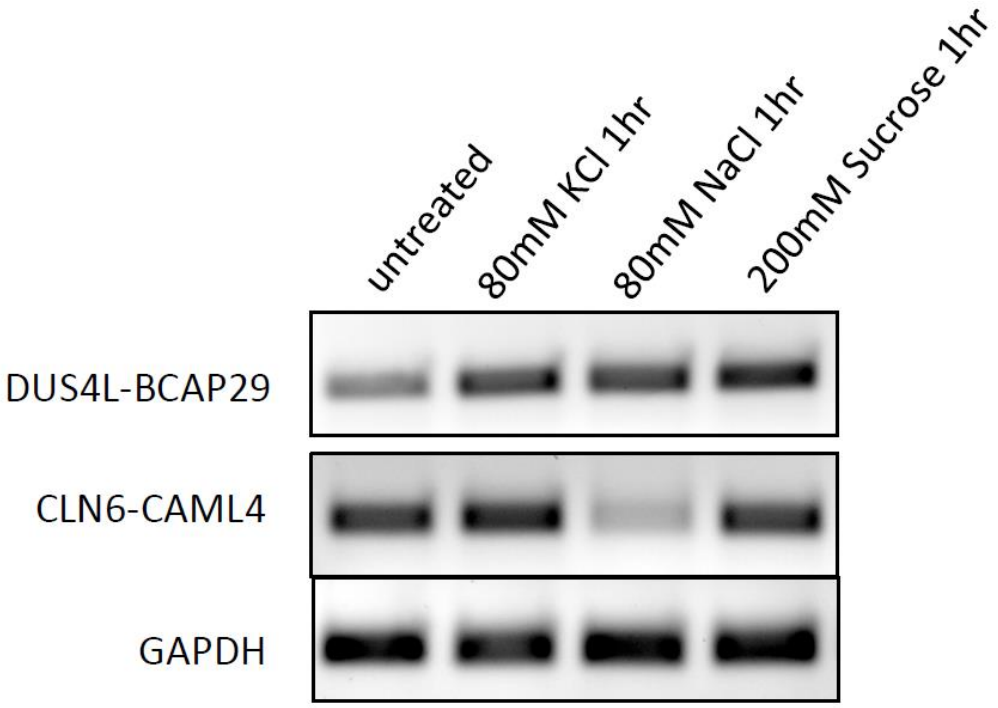

Figure S2: Gel-electrophoresis picture of representative DoGs.

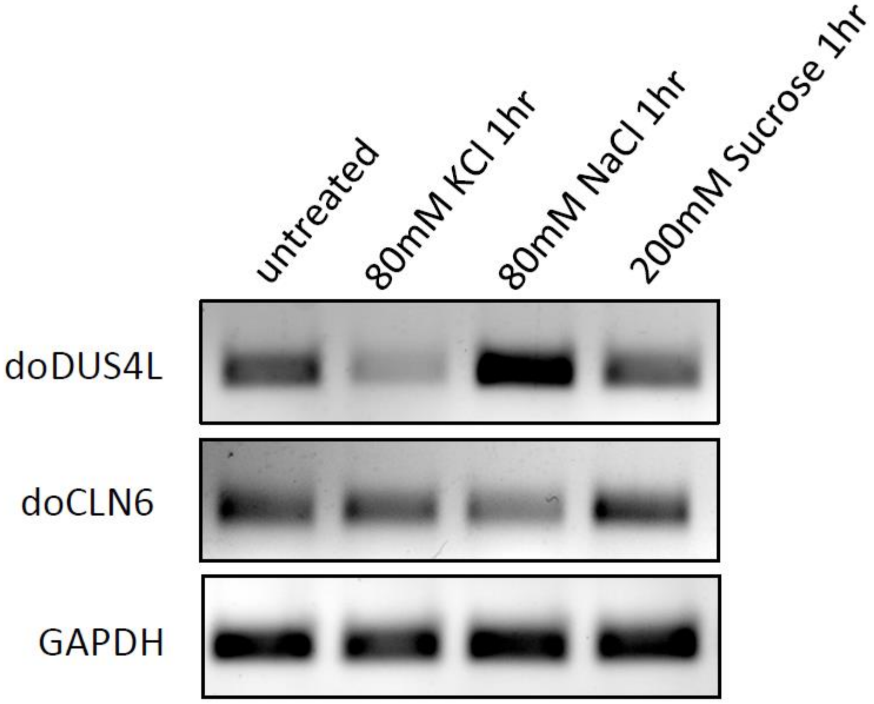

Table S1. Primer Sequences

Gene name	Forward Primer	Reverse Primer
doCLN6	gcctcaaactctgacaggga	ctttgcacacaggcgcatag
doCTNNBIP1	agacacacatccaagggtcc	tccggtttccaagctctctg
doCTSC	tcggctgacacatgagaaca	tctgacttccatgccagttag
doDUS4L	acccacctttcgtaccagtc	taaagggccaggatgaggtg
doSLC29AB1	cagtctgcagtgctgtttgg	ggcaggaggtggtaaagcaa
doUBA2	tccaagctttccctaggtgc	gctggaggtggaaagaagct
CTNNBIP1-CLSTN1	attcagcagaaggtccgagt	tgtgactatgccgtggtagg
DUS4L-BCAP29	gccagtgcactatgattcca	ggaggaataaaaggtaggcagaa
CLN6-CALML4	ttccacctcgacctctggtt	ccaccatcaacatggctaga
SLC29A1-HSP90AB1	tgttgaggtcaagtccagca	ctctccatggtgcacttcct
UBA2-WTIP	ttgaagttgttggtgatgccccg	cacacgagtcgcaggtgaagc
CTSC-RAB38	cctgtggcagctgctactcatttg	actgcttcaaatgtggctggcc
